# Relapsed multiple myeloma demonstrates distinct patterns of immune microenvironment and malignant cell-mediated immunosuppression

**DOI:** 10.1038/s41408-021-00440-4

**Published:** 2021-03-01

**Authors:** Alissa Visram, Surendra Dasari, Emilie Anderson, Shaji Kumar, Taxiarchis V. Kourelis

**Affiliations:** 1 Division of Hematology, Department of Medicine, Mayo Clinic, Rochester, MN USA; 2 University of Ottawa, Ottawa Hospital Research Institute, Ontario, Canada; 3 Department of Health Sciences Research, Mayo Clinic, Rochester, MN USA

**Keywords:** Bone marrow, Tumour immunology, Immunotherapy

## Abstract

Immunotherapy has shown efficacy in relapsed multiple myeloma (MM). However, these therapies may depend on a functional tumor immune microenvironment (iTME) for their efficacy. Characterizing the evolution of the iTME over the disease course is necessary to optimize the timing of immunotherapies. We performed mass cytometry, cytokine analysis, and RNA sequencing on bone marrow samples from 39 (13 newly diagnosed [NDMM], 11 relapsed pre-daratumumab exposure [RMM], and 13 triple-refractory [TRMM]) MM patients. Three distinct cellular iTME clusters were identified; cluster 1 comprised mainly of NDMM and RMM patients; and clusters 2 and 3 comprised primarily of TRMM patients. We showed that naive T cells were decreased in clusters 2 and 3, cluster 2 was characterized by increased senescent T cells, and cluster 3 by decreased early memory T cells. Plasma cells in clusters 2 and 3 upregulated E2F transcription factors and MYC proliferation pathways, and downregulated interferon, TGF-beta, interleuking-6, and TNF-αlpha signaling pathways compared to cluster 1. This study suggests that the MM iTME becomes increasingly dysfunctional with therapy whereas the MM clone may be less dependent on inflammation-mediated growth pathways and less sensitive to IFN-mediated immunosurveillance. Our findings may explain the decreased sensitivity of TRMM patients to novel immunotherapies.

## Introduction

Despite the advent of novel therapeutic options for treatment of multiple myeloma (MM), this disease remains incurable. While the prognosis of patients continues to improve over time^1^, numerous studies have shown that outcomes after relapse are poor^2–4^. Patients who are triple refractory (those progressing after treatment with a proteasome inhibitor (PI), an immunomodulatory drug (IMID), and an anti-CD38 monoclonal antibody) have particularly poor outcomes^5^. Tumor intrinsic and extrinsic factors drive disease refractoriness. MM is a disease comprising of multiple subclones that are genetically heterogeneous. Therapy-induced clonal selection and clonal evolution^6–8^ play a role in disease progression. In addition to changes in the plasma cell clone, alterations of the normal bone marrow (BM) immune microenvironment (iTME) can lead to tumor escape from immunosurveillance^9–12^. The importance of leveraging the immune system to control MM progression is highlighted by the recent use of immune-based therapies in heavily pretreated MM patients. These immune-based therapies rely on a functional iTME for their efficacy. However, the current paradigm is that immune effector cells used for cellular therapies (i.e., chimeric antigen receptor T cells [CAR-T]) are harvested after patients have failed numerous lines of immune-modulating therapies which drastically reshape the cellular composition of their immune system and may select for more aggressive clones. Therefore, while immunotherapeutic approaches have shown promising results^13–16^, it is not surprising that they are less effective in patients with more heavily treated triple-refractory disease^14,17,18^.

Understanding the transcriptomic (tumor) and “immunomic” landscape of relapsed myeloma is necessary in order to understand the drivers of disease progression and refractoriness to existing therapies. Characterizing the iTME may also inform decisions regarding the optimal timing to harvest immune effector cells for cellular therapies, and the ideal sequencing of immune-based therapies. Therefore, the aim of this study was to use a multiomics approach to characterize the malignant plasma cells transcriptome as well as the humoral iTME (cytokines), and cellular iTME in patients with newly diagnosed, relapsed, and triple-refractory MM.

## Methods

The Mayo Clinic biospecimen database was searched for newly diagnosed multiple myeloma (NDMM) patients, patients refractory to a proteasome inhibitor (PI) and/or an immunomodulatory drug (IMID) but not refractory to an anti-CD38 monoclonal antibody (RMM cohort), and patients refractory to a PI, IMID, and an anti-CD38 monoclonal antibody (daratumumab, no patients treated with isatuximab were identified) (TRMM cohort). All patients except one had the following bone marrow (BM) sample types: CD138+ sorted plasma cells, CD138− sorted or unsorted mononuclear cells, and BM plasma. One patient did not have CD138− cells available and was excluded from mass cytometry analysis. At our institution, patients undergo a routine BM biopsy approximately 2–3 months post autologous stem cell transplantation in order to assess disease response. However, post-transplantation BM samples were excluded given the expected significant perturbations in the iTME at this timepoint. The electronic medical record was reviewed to obtain clinical characteristics and treatment information for included patients. All included patients had consented to have their BM samples and clinical data used for research purposes, and this study was approved by the Mayo Clinic Institutional Review Board.

### Luminex analyses

Cytokine and chemokine protein levels in BM plasma were measured using Luminex xMAP technology. The multiplexing analysis was performed using the 65-plex immune monitoring kit (ProcartaPlex™, Thermofischer) on the Luminex 100 system. Raw data were analyzed per the manufacturer’s protocol. The following proteins were analyzed: cytokines [G-CSF, GM-CSF, IFN alpha, IFN gamma, IL-1 alpha, IL-1 beta, IL-2, IL-3, IL-4, IL-5, IL-6, IL-7, IL-8 (CXCL8), IL-9, IL-10, IL-12p70, IL-13, IL-15, IL-16, IL-17A (CTLA-8), IL-18, IL-20, IL-21, IL-22, IL-23, IL-27, IL-31, LIF, M-CSF, MIF, TNF alpha, TNF beta, TSLP]; chemokines [BLC (CXCL13), ENA-78 (CXCL5), Eotaxin (CCL11), Eotaxin-2 (CCL24), Eotaxin-3 (CCL26), Fractalkine (CX3CL1), Gro-alpha (CXCL1), IP-10 (CXCL10), I-TAC (CXCL11), MCP-1 (CCL2), MCP-2 (CCL8), MCP-3 (CCL7), MDC (CCL22), MIG (CXCL9), MIP-1 alpha (CCL3), MIP-1 beta (CCL4), MIP-3 alpha (CCL20), SDF-1 alpha (CXCL12)]; growth factors/regulators [FGF-2, HGF, MMP-1, NGF beta, SCF, VEGF-A; soluble receptors: APRIL, BAFF, CD30, CD40L (CD154), IL-2R (CD25), TNF-RII, TRAIL (CD253), TWEAK].

### Mass cytometry

Our antibody panel included 36 antibodies directed against well-characterized surface markers of lymphoid cells (T cells, B cells, NK− cells). Mass cytometry antibody–metal conjugate combinations are detailed in Supplementary Table 1. Sample preparation, antibody staining, and CyTOF acquisition are detailed in the Supplemental methods. Major lymphoid cell phenotypes are defined in Supplementary Table 2.

### Mass cytometry data analysis

Flow cytometry standard (FCS) files were normalized and concatenated using the Fluidigm acquisition software. FCS files were uploaded to the Astrolabe Cytometry Platform (Astrolabe Diagnostics, Inc.) where transformation, cleaning (doublets, debris), labeling, and unsupervised clustering (based on FlowSOM^19^) were done as previously described^20^. Data were transformed using arcsinh with a cofactor of 5 and the marker intensities presented in the paper are all after transformation. A minimum of 40395 CD45+ events per file were used for clustering. Since the astrolabe platform is designed to overcluster data, astrolabe-identified cell clusters were sometimes merged so that the minimum median cluster size was not smaller than the median doublet rate. As an additional method of robust cluster identification, we only considered clusters that formed discrete islands after viSNE visualization, a method that is sensitive to outliers and batch effects^21^. Identified clusters were then exported for downstream statistical analyses.

### RNA isolation and sequencing

RNA was extracted from CD138+ plasma cells using Qiagen’s RNeasy mini kit. Total RNA concentration and quality were determined using Qubit fluorometry (Invitrogen) and the Agilent Fragment Analyzer. Using the Illumina TruSeq® RNA Exome Library Prep kiT, libraries were prepared according to the manufacturer’s instructions. The concentration and purity of cDNA libraries were checked using the Agilent TapeStation D1000. Coding regions of the transcriptome were captured by pooling four of the cDNA libraries at 200 ng each. The concentration and size distribution of the completed libraries were determined using an Agilent BioAnalyzer DNA 1000 chip and Qubit fluorometry (Invitrogen). Libraries were sequenced at up to eight samples per lane following Illumina’s standard protocol using the Illumina cBot and HiSeq 3000/4000 PE Cluster Kit. The flow cells were sequenced as 100 × 2 paired-end reads on an Illumina HiSeq 4000 using the HiSeq Control Software HD 3.4.0.38 collection software. Base-calling was performed using Illumina’s RTA version 2.7.7.

### Statistical methods

Hierarchical clustering of patients according to the abundance (% of CD45+ cells) of immune subsets was performed using the Ward method. The Kruskal–Wallis statistical test was used to describe differences between groups. Kaplan–Meier survival analysis was used to estimate the overall survival (defined as time between sample collection and death or last follow-up) and progression-free survival (defined as time between sample collection and death/progression or last follow-up). Pearson’s correlation was used to test correlations between continuous variables. A two-sided false discovery rate (FDR) adjusted *p* value of < 0.05 was considered significant. Statistical analysis was performed using JMP Pro statistical software version 14.1 (SAS Institute, Cary, NC). Differential gene expression across groups of interest was performed using the edgeR software. Gene set enrichment analysis (GSEA)^22^ using the hallmarks of cancer database, was performed to identify differentially expressed (FDR < 0.05) gene pathways between groups.

## Results

### Patient characteristics

A total of 39 (13 NDMM, 11 RMM, and 15 TRMM) patients were included in this study. Their clinical and laboratory characteristics are shown in Supplementary Table 3. As expected, triple-refractory (TRMM cohort) patients had worse OS (median 12.4 [95% CI 6.1–15.5] months) and PFS (6.1 [95% CI 5.4–19.1] months) from sample collection than the RMM (median OS 124.1 [95% CI 25.9–131] months) and NDMM (median 110.3 [95% CI 19.3–132.4] months) cohorts. However, the NDMM cohort in this study tended to have an unusually aggressive disease course (Supplementary Fig. 1); the PFS from diagnosis was ≤12 months in 8/13 (64%) of NDMM patients, despite the fact that all patients received novel agent induction regimens, and 10/13 (77%) received triplet or quadruplet induction regimens.

### Triple-refractory patients have a distinct cellular iTME

Given the small size and heterogeneity of our cohort, and to allow a less biased (by prior knowledge of patients’ relapse status) analytic approach, we agnostically grouped patients based on their cellular iTME composition and use that as our “anchoring variable” for several reasons. First, the efficacy of novel immunotherapies, which largely target plasma cells irrespective of their clonal biology, depends on a functional cellular iTME. Second, the humoral iTME (cytokines) is much less stable over time compared to the individual cellular composition, and so grouping based on cytokine expression may be less reliable. Furthermore, sample cytokine profiles are likely reflective of the cellular microenvironment, which was captured by our mass cytometry and transcriptomic data. Finally, our cohort was small and heterogeneous, as was evident by the unusually aggressive disease course of NDMM patients. For this reason, we did not use transcriptomic-based grouping or grouping based on a priori knowledge of their relapse status (NDMM, RMM, TRMM).

**Fig. 1:**
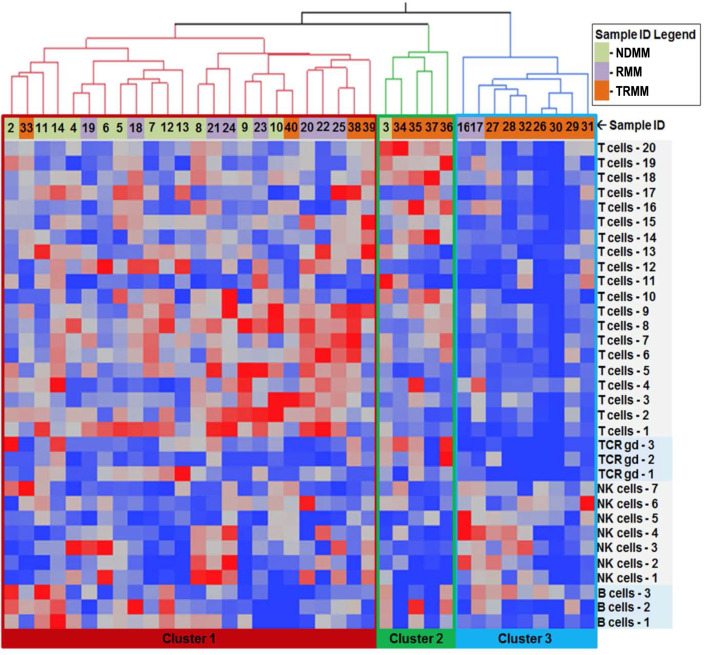
Hierarchical clustering of patients based on immune subset frequencies (% of total CD45+ cells) identifies three distinct immune clusters. Samples represent patients with newly diagnosed MM (NDMM), relapsed MM not refractory to anti-CD38 antibodies (RMM), and relapsed MM that are triple refractory (TRMM)

**Fig. 2:**
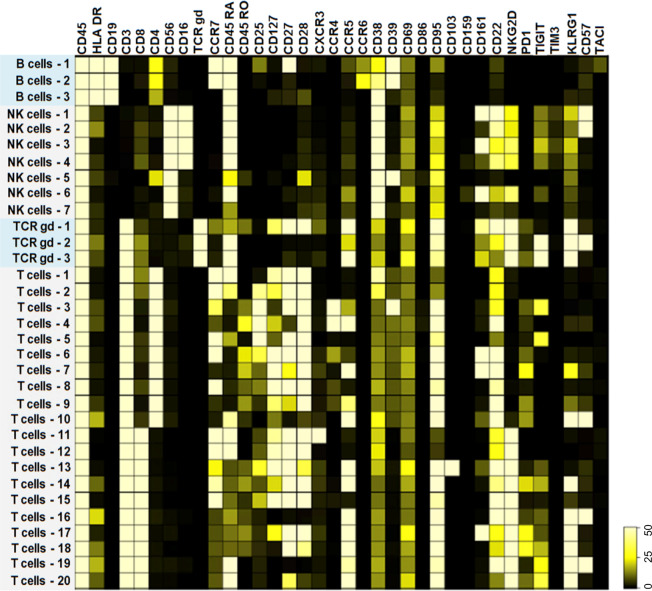
Immune phenotype of cellular subsets. The heat map highlights the marker expression within each unique cellular subset identified by mass cytometry

Cellular iTME-based hierarchical clustering recapitulated disease biology based on prior treatment exposure very accurately (Figs. 1 and 2) and identified three distinct groups: Cluster 1 comprised mainly of NDMM and RMM patients, whereas clusters 2 and 3 were comprised primarily of TRMM patients. The clinical characteristics of patients based on immune clusters are summarized in Table 1. Patients in cluster 2 were mostly (4/5 patients) triple refractory but less heavily pretreated compared to cluster 3 (2 median lines of therapy for cluster 2 versus 8 lines for cluster 3, *p* < 0.001). The median overall survival (mOS) from sample collection was significantly better for patients in cluster 1 (Supplementary Fig. 2). However, 4 of the 5 patients in cluster 2 had their samples collected while relapsing on daratumumab therapy. In order to ensure that recent daratumumab exposure was not the main factor altering the immune microenvironment and driving the cellular iTME clustering, we repeated the cellular iTME hierarchical clustering after excluding all seven patients that had samples collected while progressing on daratumumab-based therapy. Only two patients (both with NDMM) were clustered differently. Overall, the composition of patients within the cellular iTME clusters was similar with or without the inclusion of patients progressing on daratumumab (cohen’s kappa coefficient = 0.863, with *p* < 0.001), suggesting that the iTME clustering was not driven primarily by recent daratumumab exposure. These data suggest that triple-refractory patients have a distinct cellular iTME compared to non-triple-refractory patients.

**Table 1:** Characteristics of multiple myeloma grouped by the distinct immune cell clusters.

	Cluster 1 (*n* = 24)	Cluster 2 (*n* = 5)	Cluster 3 (*n* = 9)	*P* value
Median age at sample collection—years (IQR)	59 (48–68)	66 (60–67)	59 (52–67)	0.25
Disease status				<0.001
NDMM—*n* (%)	12 (50%)	1 (20%)	0 (0%)	
RMM—*n* (%)	8 (33%)	0 (0%)	2 (22%)	
TRMM—*n* (%)	4 (17%)	4 (80%)	7 (78%)	
Cytogenetic risk at sample collection				0.118
Standard risk—*n* (%)	14 (58%)	1 (20%)	6 (67%)	
High risk^a^—*n* (%)	8 (33%)	4 (80%)	2 (22%)	
Missing^b^—*n* (%)	2 (8%)	0 (0%)	1 (11%)	
Prior therapy				
ASCT—*n* (%)	5 (21%)	2 (40%)	8 (89%)	<0.001
PI refractory—*n* (%)	6 (25%)	4 (80%)	9 (100%)	<0.001
IMID refractory—*n* (%)	11 (46%)	4 (80%)	9 (100%)	0.011
Anti-CD38 antibody refractory—*n* (%)	4 (17%)	4 (80%)	7 (78%)	0.001
Median time between anti-CD38 exposure and sample collection—months (range)	1.5 (0–18.9)	0 (0–0)	2.8 (0–17.3)	0.133
Median time between diagnosis and sample collection—months (range)	0 (0–1.4)	8.5 (3.5–82.5)	71.4 (27.6–82.5)	<0.001
Median lines of therapy prior to sample collection—*n* (IQR)	0 (0–2)	2 (1–7)	8 (4–9)	<0.001

^a^High risk FISH is defined as the presence of del(17p), t(4;14), or t(14;16).

^b^FISH at sample collection was not available, however, all of these patients had standard-risk myeloma at diagnosis.

### Triple-refractory relapses are associated with shrinking of the CD4 T-cell pool and increased terminally differentiated T cells

We then considered lymphoid cell subset differences across the three clusters. We noted no differences in B cell subset frequencies between the three immune clusters. There was also no significant difference in the overall NK-cell infiltration between the clusters. However, one CD39+/NKG2D− NK-cell subset (NK cell-5), which is thought to have immunosuppressive properties^23,24^ and unable to recognize MM cells^25^, was increased in cluster 3.

When considering T-cell differences, the overall T-cell infiltration was significantly lower in cluster 3 patients compared to clusters 1 and 2 (23% vs 40% vs 51%, respectively, *p* < 0.001). The CD4 to CD8 ratio was significantly higher in cluster 1 compared to clusters 2 and 3 (2.0 vs 0.61 vs 1.15, respectively, *p* < 0.001). CD4+ T cells were highest in cluster 1 compared to 2 and 3 (median 26.97% of CD45+ cells versus 16.3% and 7.9%, respectively, *p* < 0.0001). However, CD8+ T cells were highest in cluster 2 (median 18.4%, 36.3%, and 9.5% of CD45+ cells for clusters 1, 2, and 3, respectively, *p* < 0.0001).

**Fig. 3:**
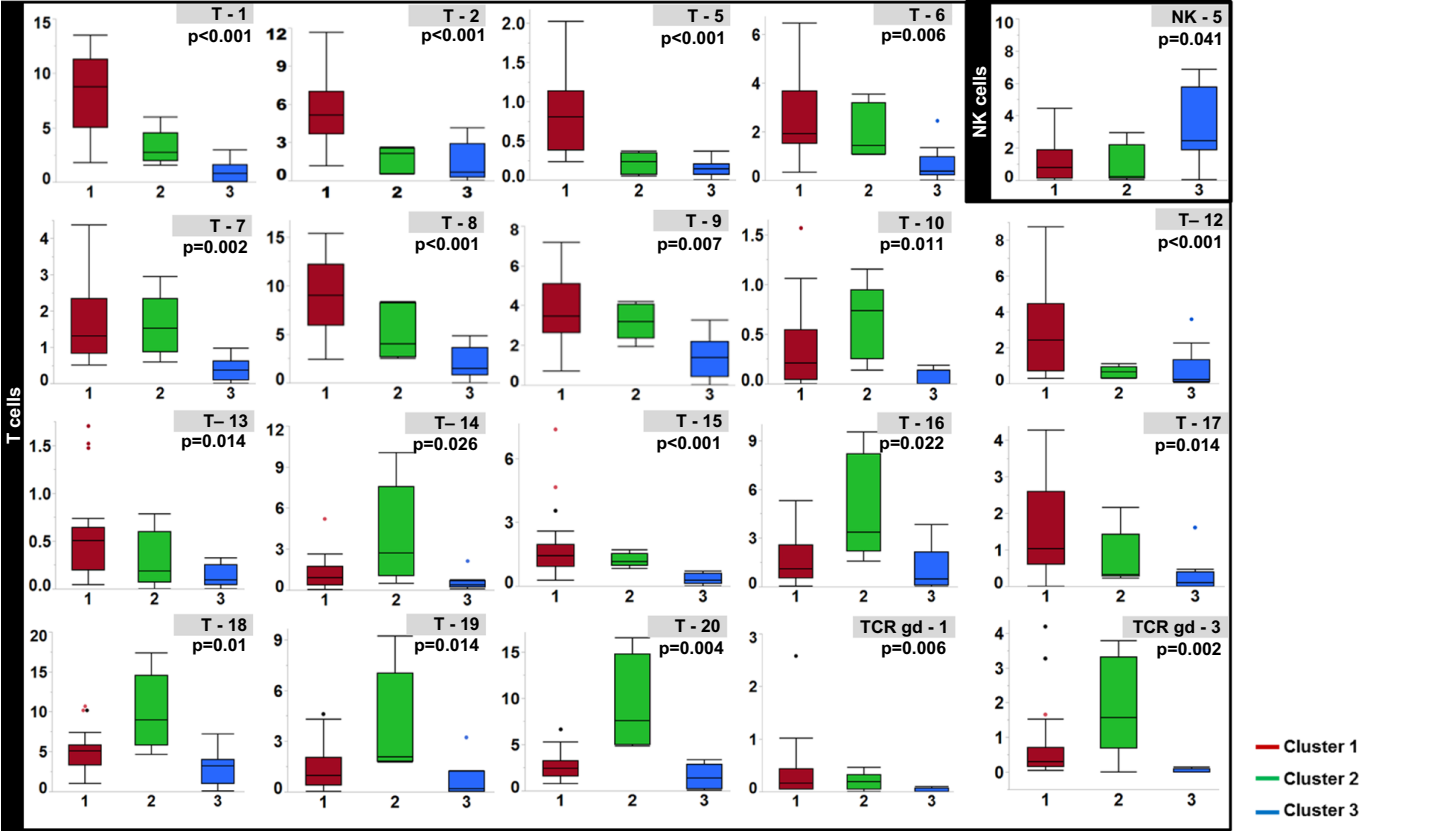
Box plots showing the differential abundance of immune subsets. The frequency of immune cell subsets within each iTME cluster are shown as the percentage of total CD45+ cells. For clarity, only subsets that were significantly different between (false discovery rate corrected *p* value of < 0.05) the three clusters are shown

**Fig. 4:**
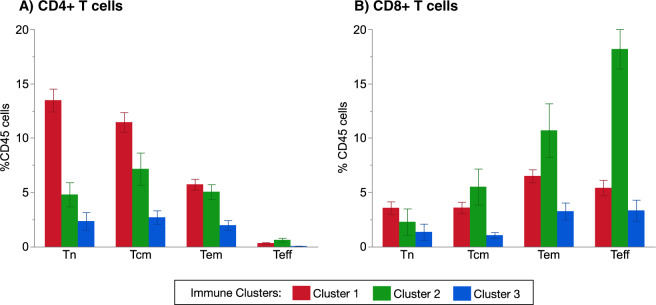
Differential abundance of T-cell phenotypes within each patient cluster. CD4+ T-cell subsets are shown in (**A**), with the percentages of Tn (naïve T cells, comprising subsets T1 and T2), Tcm (central memory T cells, comprising of subsets T6 and T8), Tem (effector memory T cells, comprising of subsets T4, T7, T9), and Teff (effector T cells, comprising of the T10 subset). CD8+ T-cell subsets are shown in (**B**), with the percentages of Tn (naive T cells, comprising subsets T11 and T12), Tcm (central memory T cells, comprising of subsets T13, T14, T15), Tem (effector memory T cells, comprising ofsubsets T17, T18), and Teff (effector T cells, comprising of the subsets T16, T19, T20). Results are presented as mean ± standard error

The differential abundance of specific immune subsets is shown in Fig. 3. Cluster 1 was characterized by an increased abundance of the T1, T2, and T12 populations which had phenotypes consistent with naïve CD4 and CD8 T cells, as shown in Fig. 4. The CD8/CD161+ T17 subset was also highest in cluster 1. Other CD4/CD161+ T-cell populations (T6, T7) were lowest in cluster 3 but preserved in cluster 2. CD161+ T cells are thought to represent T-cell subsets with similar transcriptional and phenotypic features, high proliferative capacity, and the ability to secrete high levels of IFN gamma and TNF alpha^26,27^. Cluster 2 was characterized by a higher abundance of several immunosenescent, terminally differentiated (CD27/CD28−, CD57/KLRG1+/−) populations (T10, T16, T18, T19, T20, TCRgd-3) as well as the T14 population characterized by tissue homing (CCR5+) and early differentiation markers (CD27/28+) but also exhaustion markers (PD-1, TIGIT). Cluster 3 was characterized by a decrease of the following subsets: the T8 and T15 populations, with a central memory (CCR7/CD127+) phenotype; the CCR5+ T9 effector memory population, a subset necessary for maximal T-cell-mediated antitumor responses^28^; the CD103+ T13 population, thought to consist of tumor-reactive, tissue-resident memory T cells^29,30^; and the TCRgd-1 subset expressing markers of early differentiation (CD27, CD28), tissue homing (CCR5), and tumor cytotoxicity (CD226)^31^.

These data suggest that triple refractoriness is associated with shrinking of the T-cell pool overall, especially CD4+ and naïve subsets. Consequently, within some triple-refractory patients, early memory T-cell subsets decrease and in others senescent, terminally differentiated T-cell subsets accumulate.

### Triple-refractory relapses are associated with proliferative plasma cell clones that downregulate immune effector molecule signaling pathways

**Fig. 5:**
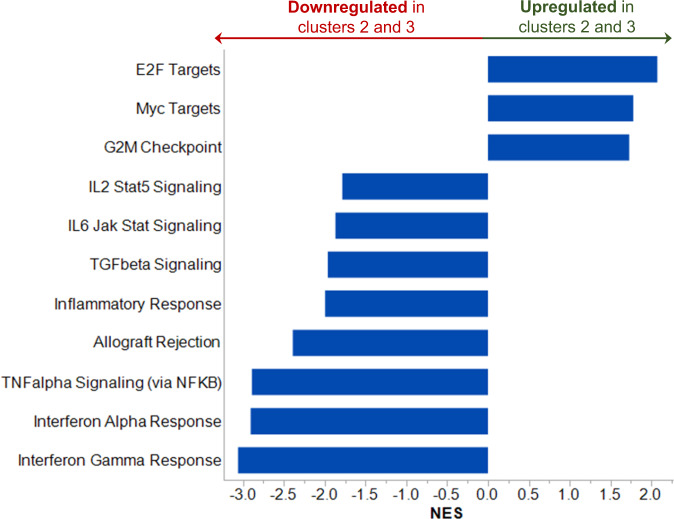
The mean normalized enrichment score (NES) of Hallmark pathways with FDR *q* value < 0.05 when comparing gene expression in the immune clusters 2 and 3 versus cluster 1 are represented by each bar. NES < 0 represents downregulation of specified pathway in immune clusters 2 and 3, versus cluster 1. NES > 0 represents upregulation of specified pathway in immune clusters 2 and 3, versus cluster 1

Since patients in clusters 2 and 3 above, enriched in TRMM cases, had a distinct iTME compared to cluster 1 (comprised primarily of NDMM and RMM patients), we performed GSEA of the malignant plasma cells and compared cluster 1 to the combined cluster 2 and 3 groups. In patients from clusters 2/3, E2F targets, G2M checkpoint genes, and Myc targets were increased (Fig. 5), suggesting that malignant cells from clusters 2/3 upregulated transcriptional networks related to DNA replication, cell division, and MYC signaling. This group also showed downregulation of various inflammatory pathways, most notably tumor necrosis factor alpha (TNFa) and interferon (IFN) signaling. Of note, we found no cytokines differentially expressed between the three immune clusters identified above or when considering clusters 2 and 3 together. For this reason, we do not present the cytokine data that can be made available upon request. These data suggest that more heavily pretreated patients have highly proliferative malignant clones that downregulate pathways required for tumor immunosurveillance.

## Discussion

In this study, we evaluated the humoral and cellular iTME and malignant clone of patients with NDMM, RMM, and TRMM. We found that most triple-refractory patients had a distinct iTME characterized by reduction of their CD4 T-cell pool and lower levels of naive T-cell subsets. Within most triple-refractory patients, central memory T cells were decreased along with several other subsets involved in antitumor immunity. We showed that the malignant plasma cells of triple-refractory patients are highly proliferative, as expected, but also demonstrate downregulation of several signaling pathways implicated in immune-mediated tumor rejection.

Most of the lymphoid compartment iTME differences in our cohort were driven by T cells. This could be due to the T-cell-focused marker selection in our mass cytometry panel. It could also be a result of B cell immunoparesis that is present at diagnosis or relapse irrespective of treatment status^32,33^. Interestingly, NK cells, which are uniformly CD38+ and decrease after treatment with daratumumab^34^, were preserved. This is in agreement with prior data that suggest that the NK cell pool recovers within 3–6 months from discontinuing daratumumab^34^ and suggests that therapies that rely on NK-cell-mediated cytotoxicity, such as elotuzumab^35^, could be employed early after CD38 antibody relapse. However, NK cells lacking activating receptors required for antitumor responses, such as NKG2D^36^ and CD226^37^, were increased in most triple-refractory patients which could explain the decreased efficacy of NK-cell-directed therapy in these patients.

Most non-triple-refractory patients (cluster 1) had higher levels of CD4+ T cells and subsets with a naïve phenotype. For NDMM within cluster 1, this could reflect the inability of the T-cell compartment to mount a cytotoxic response, which would require more differentiated CD8+ effector cells, against myeloma. For RMM within cluster 1, this likely reflects the differential effects of prior therapies on the iTME compared to anti-CD38 antibody-treated patients. For instance, lenalidomide therapy increases the proportion of less differentiated T cells, including naïve T cells^38,39^. On the contrary, therapy with anti-CD38 monoclonal antibodies, skews the T-cell pool toward more differentiated T-cell phenotypes^40^ with more effector functions. These effector cells are largely responsible for the efficacy of CD38 antibodies since they clonally expand to eradicate malignant cells^41^. Alternatively, since the T1 and T2 naive subsets were both CD38+, daratumumab could have caused “on-target off-tumor” depletion of these subsets.

Once the disease relapses after anti-CD38 antibody therapy, the accumulation of terminally differentiated T-cell subsets and the decrease in naïve and early memory phenotypes leaves triple-refractory patients with a dysfunctional T-cell compartment. This effect was more pronounced in cluster 2, which included 4 of 5 patients relapsing on daratumumab. Many CAR-T-cell products are currently manufactured from cells harvested at the time of relapse, and so for triple-refractory patients in this study their apheresis product would consist of lower levels of CD4+ and early memory T cells. However, both a balanced CD4:CD8 ratio as well as less differentiated T-cell phenotypes are associated with superior efficacy of CAR-T products^18,42–44^. Therefore, optimizing the timing of T-cell collection in order to maximize naïve and memory T-cell subsets, and minimize terminally differentiated, senescent, or exhausted T-cell subsets, may improve the efficacy of cellular therapies in multiple myeloma^43,44^. For example, T cells harvested after induction therapy may have a higher CD8+ memory phenotype^18^, whereas T cells collected early after autologous stem cell transplant have been associated with higher levels of differentiated T cells^45^. Expansion of CAR-T cells with IL-7 can lead to superior CAR-T-cell expansion and long-term antitumor activity, and could be an alternative strategy to optimize CAR-T proliferation considering the expression of IL7R/CD127 in several T-cell subsets in our study^46^. Finally, even in relapsed patients not receiving CAR-T cells, the ability of the existing T-cell pool to differentiate and expand in response to other novel monoclonal antibodies will likely be limited, which may explain the poor responses to therapy in heavily pretreated patients.

While the state of the cellular iTME presents one challenge for novel immunotherapies, the characteristics of the malignant clone cannot be ignored. Here we found that heavily pretreated patients (clusters 2 and 3) upregulated gene pathways associated with proliferation. This was not unexpected and should not be a specific barrier for the efficacy of novel immunotherapies that target malignant cells irrespective of their proliferative capacity. However, we also showed that malignant cells from these patients had adapted to downregulate gene pathways associated with immune-mediated tumor rejection, such as IFN. Deletion of IFN and TNF signaling has been shown to protect tumor cells from T-cell-mediated killing, thereby providing a mechanism for tumors to evade the immune system^47^. We also showed that MM cells at this stage may be less dependent on inflammation-mediated growth pathways such as IL-6 and TGF-beta. Although the mechanisms behind these observations are unclear, anti-CD38 antibody-mediated clonal selection of malignant cells able to evade immune effector cells and with more proliferative characteristics is likely implicated. This finding is relevant for the use of novel immunotherapies, including CAR-T cells, which can induce cytokine signaling after stimulation^48^, and therapeutic antibodies^35^ that depend partially on intact tumor IFN and TNF mediated signaling for T-cell-mediated tumor eradication^47,49^.

Interestingly, we did not find any significant differences in the humoral iTME (cytokines) according to relapse status (NDMM//RMM/TRMM) or immune clustering. This could be due to the small size and heterogeneity of our cohort. Furthermore, since all our samples were obtained when the disease was active (at diagnosis/relapse), it is possible that all the known inflammatory cytokines associated with MM growth were uniformly elevated.

Our study has some important limitations. Our NDMM had a median time to progression from first-line therapy of <20 months, likely reflecting selection bias of patients who were referred to our institution at diagnosis and had all necessary biological samples prior to initiating induction therapy. Due to limitations in sample availability, we were unable to study paired samples collected from uniformly treated patients over the course of their disease, or samples matched based on prior treatment exposure. At our institution, we do not routinely obtain BM samples with every relapse. Therefore, the heterogeneity in treatments is a major limitation of the study and precludes our ability to ascertain whether the observed aberrations in the iTME were a result of specific therapies (e.g., anti-CD38 antibody refractoriness) or, more likely, were secondary to the cumulative effects of all prior therapies and the proliferation of aggressive malignant clones. Future work should focus on validating these results in a cohort of patients with paired samples before and after anti-CD38 antibody refractoriness develops. Furthermore, our study did not report on alterations in the myeloid cell compartment. Our mass cytometry panel did not characterize myeloid cells, and the limited availability of sample material restricted the maximum number of CD45+ cells we could analyze and, consequently, the resulting analytical depth (smallest detectable immune subset). Furthermore, whereas lymphoid cell loss occurs in a stochastic manner in cryopreserved samples, the same is not true for several significant myeloid subsets, such as myeloid-derived suppressor cells, that are preferentially lost with cryopreservation^50^.

In summary, here we have demonstrated that triple-refractory MM patients have a distinct cellular iTME in addition to malignant clone characteristics that could make them less suitable candidates for novel immunotherapies. Despite that, durable responses are noted in triple refractory patients treated with CAR-T cells, bispecific antibodies, and antibody–drug conjugates. However, our results suggest that utilizing these therapies earlier in the disease course, and in combination with existing novel agents and immunotherapies may be more appropriate. Harvesting of T cells for CAR-T-cell generation should also be performed earlier in the disease course in order to maximize naïve and central memory T-cell subsets in the CAR-T product, and this may lead to more effective and durable responses.

## Supplementary information

Supplemental material

Reproducibility checklist
