# Supplementary material for: Relapsed multiple myeloma demonstrates distinct patterns of immune microenvironment and malignant cell-mediated immunosuppression
Source: Blood Cancer J. 2021 Mar 1;11(3):45. doi: 10.1038/s41408-021-00440-4 (PMC7921408; doi:10.1038/s41408-021-00440-4)
Supplement: Supplementary file 1 — Supplemental material [file 41408_2021_440_MOESM1_ESM.pdf]

Supplemental Methods

Antibody and sample preparation

For primary conjugations, purified antibodies were obtained in carrier protein-free PBS and labeled using the X8 polymer MaxPAR antibody conjugation kit (Fluidigm) according to the manufacturer's protocol. The antibody panel was designed using the web-based Fluidigm panel designer to select channels with optimal signal and minimal background from oxidation, isotopic impurity or abundance sensitivity. All antibodies were titrated to optimal staining concentrations using primary human bone marrows of patients with MM. Antibody master mixes were prepared fresh for each experiment. A reference sample was run with each experiment to evaluate for staining inconsistencies. All BM samples were processed identically. Mononuclear cells were obtained after ACK lysis of BM samples and were viably frozen in RPMI 1640, 20%FBS, 10% DMSO. Cryopreserved cells were resuscitated for mass cytometry analyses by rapid thawing and were rested in RPMI 1640 (20% FBS) for 60 minutes prior to staining. Staining was performed using Fluidigm's protocol. Briefly, 1-3 million cells were stained for viability with 5mM cisplatin for 5 mins at room temperature and quenched with cell staining medium (CSM, Fluidigm). Cells were then incubated for 10 mins at room temperature with human FcR blocking reagent (Biolegend) and then stained with the surface antibody cocktail for 60mins at 4°C with gentle agitation. Finally, cells were washed twice with CSM, fixed with 1.6% PFA, washed with CSM and resuspended in 1:1000 solution of Iridium intercalator diluted in MaxPar Fix and Perm buffer (Fluidigm) for 20mins at room temperature. Prior to acquisition, cells were washed twice in CSM and twice in deionized water and were then diluted to a concentration 0.5million cells/ml in water containing 10% of EQ 4 Element Beads (Fluidigm). Cells were filtered through a 35μm membrane prior to mass cytometry acquisition. Samples were then acquired on a Helios mass cytometer.

Supplemental table 1. CytOF antibody panel

Target	Clone	Metal tag	Source
CD45	HI30	89Y	Fluidigm
CD196 (CCR6)	G034E3	141Pr	Fluidigm
CD19	HIB19	142Nd	Fluidigm
CD127 (IL-7Ra)	A019D5	143Nd	Fluidigm
CD38	HIT2	144Nd	Fluidigm
KLRG1	A1	145Nd	Biolegend*
CD69	FN50	146Nd	Biolegend*
CD159a (NKG2A)	Z199	147Sm	R&D systems*
CD95/Fas	DX2	148Nd	Biolegend*
CD194 (CCR4)	205410	149Sm	Fluidigm
CD134 (OX40)	ACT35	150Nd	Fluidigm
CD103	Ber-ACT8	151Eu	Fluidigm
TCRgd	11F2	152Sm	Fluidigm
TIGIT	MBSA43	153Eu	Fluidigm
TIM-3	F38-2E2	154Sm	Fluidigm
CD45RA	HI100	155Gd	Fluidigm
CD195 (CCR5)	NP-6G4	156Gd	Fluidigm
CD27	L128	158Gd	Fluidigm
TACI		159Tb	Biolegend*
CD28	CD28.2	160Gd	Fluidigm
CD279 (PD-1)	EH12.2H7	161Dy	Biolegend*
CD183 (CXCR3)	G025H7	163Dy	Fluidigm
CD161	HP-3G10	164Dy	Fluidigm
CD45RO	UCHL1	165Ho	Fluidigm
CD314 (NKG2D)	ON72	166Er	Fluidigm
CD197 (CCR7)	G043H7	167Er	Fluidigm
CD8a	SK1	168Er	Fluidigm
CD25 (IL-2R)	2A3	169Tm	Fluidigm
CD3	UCHT1	170Er	Fluidigm
CD226	DX11	171Yb	Fluidigm
CD57	HCD57	172Yb	Fluidigm
HLA-DR	L243	173Yb	Fluidigm
CD4	SK3	174Yb	Fluidigm
CD39	A1	175Lu	Biolegend*
CD56 (NCAM)	NCAM16.2	176Yb	Fluidigm
CD16	3G8	209Bi	Fluidigm

* These antibodies were conjugated to their respective metal tags using the X8 polymer MaxPAR antibody conjugation kit (Fluidigm) according to the manufacturer’s protocol.

Supplemental table 2. Definitions of major lymphoid cell phenotypes

**B Cells**	CD19+
**NK Cells**	CD56+, CD16+/dim
**T Cells**	CD3+, CD4+ or CD8+
**Naïve**	CCR7+, CD45RO-
**Central memory**	CCR7+, CD45RO+
**Effector memory**	CCR7-, CD45RO+
**Effector**	CCR7-, CD45RO-
**T regulatory cells**	CD25+, CD127-, CCR4+

Supplemental Table 3. Characteristics of multiple myeloma patients at diagnosis and at the time of sample collection

	NDMM (n=13)	RMM (n=11)	TRMM (n=15)	P value
**Diagnosis data**				
Median age - years (range)	60 (42.9-82.2)	50.7 (41.2-68.8)	59.4 (54.9-67.3)	0.463
R-ISS				0.116
1 - n (%)	3 (25)	0	5 (36)	
2 - n (%)	7 (58)	5 (83)	9 (64)	
3 - n (%)	2 (17)	1 (17)	0	
FISH				0.198
Standard risk - n (%)	8 (62)	10 (91)	8 (67)	
High risk[a] - n (%)	5 (38)	1 (9)	4 (33)	
Median follow up from diagnosis - months (IQR)	69 (16.7-122.1)	94.7 (60.9-120.7)	75.1 (56.6-126.1)	0.469
Median lines of therapy between diagnosis and last follow up - n (IQR)	6 (3.5-10)	6 (4-8)	9 (7-10)	0.199
**At sample collection**				
Median age - years (range)	60 (43-82)	58 (42-77)	69 (57-83)	0.075
Median time between diagnosis and sample collection - months (IQR)	0 (0-0.5)	8.9 (5.2-28.4)	71.4 (48.2-118.3)	<0.0001
Median prior lines of therapy - n (IQR)	0	2 (1-2)	6 (4-8)	<0.0001
FISH				0.19
Standard risk - n (%)	8 (62)	6 (86)	7 (47)	
High risk[a] - n (%)	5 (38)	1 (14)	8 (53)	
Prior therapy				
ASCT - n (%)	0	4 (36)	13 (87)	<0.0001
PI exposure - n (%)	0	7 (64)	15 (100)	<0.0001
IMID exposure - n (%)	0	11 (100)	15 (100)	<0.0001
Anti-CD38 Antibody exposure - n (%)	0	0	15 (100)	<0.0001

[a]High risk FISH is defined as the presence of del(17p), t(4;14), or t(14;16)

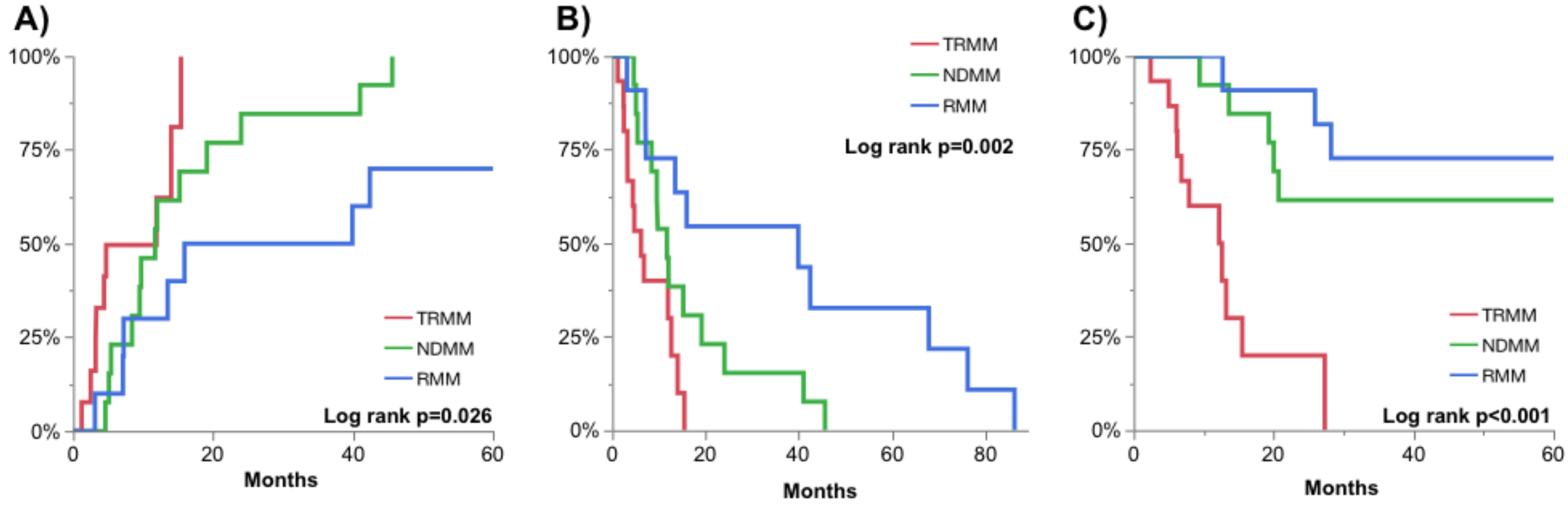

Supplementary Figure 1. Progression and survival of patients by treatment group. A) time to progression, B) progression free survival, C) overall survival. As expected, the triple refractory (DRMM) group fared consistently worse. However, this newly diagnosed (NDMM) cohort in this study had an unusually aggressive course, with a worse TTP, PFS, and OS compared to the relapsed (RMM) cohort. **NDMM:** newly diagnosed myeloma**, RMM:** relapsed myeloma, non-triple refractory, **TRMM:** triple refractory MM.

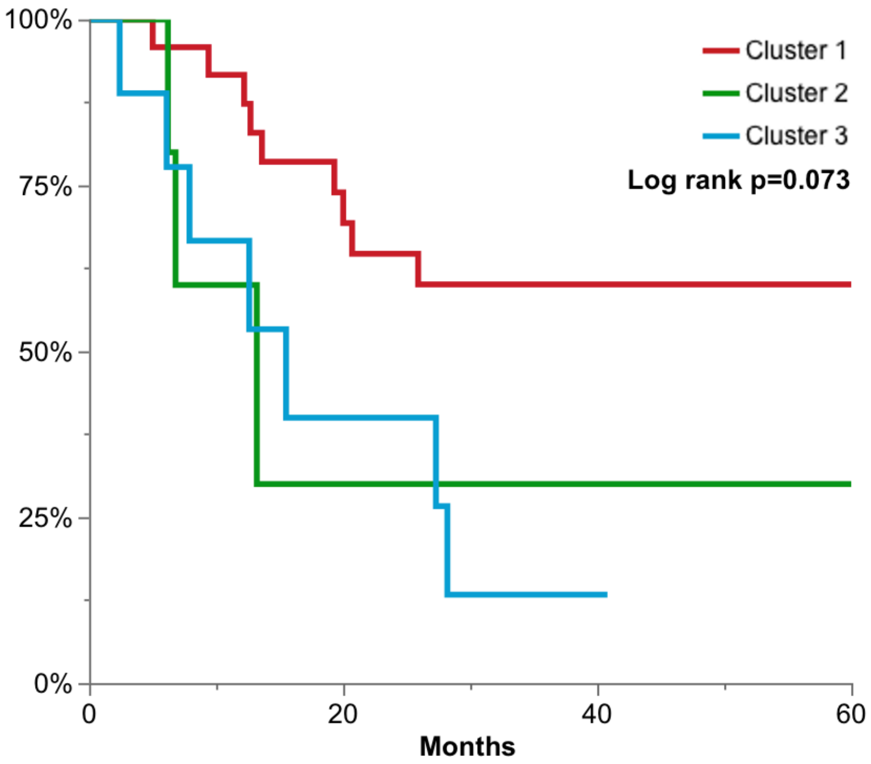

Supplementary Figure 2. Overall survival from time of the sample collection to last follow up is shown for myeloma patients, clustered by the immune subset frequencies.
